# Supplementary material for: Combining amino acid PET and MRI imaging increases accuracy to define malignant areas in adult glioma
Source: Nat Commun. 2023 Jul 29;14:4572. doi: 10.1038/s41467-023-39731-8 (PMC10387066; doi:10.1038/s41467-023-39731-8)
Supplement: Supplementary file 1 — Supplementary Information [file 41467_2023_39731_MOESM1_ESM.pdf]

Supplementary Information

Table S1. Histopathological and molecular results obtained from material collected from different target areas with visible contrast enhancement or simultaneous tracer uptake in PET along with the final histopathological result.

**Patient**	**Sex/Gender**	**T1Gad+/PET+**	**T1Gad+/PET-**	**T1Gad-/PET+**	**T1Gad-/Flair+/PET-**	**Final HP result (WHO 2021)**	***MGMT* methylation**	**Codeletion 1p/19q**	***IDH1* mutation**	***IDH2* mutation**	***Previous irradiation***	***Overall Surviva [months]l***
**1**	F	WHO III	-	WHO III	WHO II	Oligodendroglioma, IDH- mutant, G3	+	+	+	—	0	79
**2**	M	WHO III	-	WHO III	No tumor cells	Astrocytoma, IDH-mutant, G3[2]	+	—	+	—	0	52
**3**	F	WHO III	WHO II	WHO III	WHO III	Oligodendroglioma, IDH- mutant, G3	+	+	+	—	0	78
**4**	M	WHO III	-	WHO III	WHO II	Astrocytoma IDH-mutant, G3[2]	+	+	+	—	+	14
**5**	F	WHO III	-	WHO IV	No tumor cells	Glioblastoma, IDH wildtype G4	+	—	—	—	0	7
**6**	M	WHO IV	WHO IV	WHO IV	WHO IV	Glioblastoma, NOS G4	Not performed [1]	Not performed [1]	Not performed [1]	Not performed [1]	0	3
**7**	M	WHO III	-	WHO III	WHO II	Astrocytoma, IDH-mutant, G3[2]	Not performed [1]	Not performed [1]	+ [1]	Not performed [1]	0	13
**8**	M	WHO III	-	WHO IV	No tumor cells	Glioblastoma, NOS G4	Not performed [1]	Not performed [1]	Not performed [1]	Not performed [1]	0	2
**9**	M	WHO III	-	WHO III	WHO II	Oligodendroglioma, IDH- mutant, G3	Not performed [1]	Not performed [1]	+ [1]	Not performed [1]	0	64
**10**	M	WHO IV	-	WHO IV	No tumor cells	Glioblastoma, IDH wildtype G4	+	—	—	—	0	14
**11**	M	WHO II	-	WHO II	-	Astrocytoma, NOS, G2	Not performed [1]	Not performed [1]	Not performed [1]	Not performed [1]	0	51
**12**	F	WHO III	-	WHO III	WHO III	Oligodendroglioma, IDH- mutant, G3	+	+	+	—	0	59
**13**	M	WHO IV	-	WHO IV	-	Glioblastoma, IDH wildtype G4	+	—	—	—	0	37
**14**	F	WHO II	-	WHO III	No tumor cells	Astrocytoma, IDH-mutant, G3[2]	+	—	+	—	+	54

**15**	M	WHO IV	-	WHO IV	WHO IV	Glioblastoma, IDH mutant G4	+	+	+	—	+	28
**16**	F	-	-	WHO II	No tumor cells	Astrocytoma, IDH wildtype G2 [2]	—	—	—	—	0	50
**17**	F	WHO IV	-	WHO IV	-	Glioblastoma, IDH mutant G4	+	—	+	—	0	2
**18**	M	WHO IV	-	WHO IV	No tumor cells	Glioblastoma IDH wildtype G4	—	—	—	—	0	18
**19**	F	WHO IV	-	WHO IV	WHO III	Glioblastoma IDH wildtype G4	+	—	—	—	+	25
**20**	F	WHO III	-	WHO III	No tumor cells	Oligodendroglioma, IDH- mutant, G3	+	+	+	—	+	52
**21**	F	-	-	WHO III	WHO II	Oligodendroglioma, IDH- mutant,G3	+	+	+	—	0	48
**22**	F	WHO III	-	WHO IV	WHO III	Glioblastoma NOS G4	Not performed [1]	Not performed [1]	Not performed [1]	Not performed [1]	0	48
**23**	F	-	-	WHO IV	WHO III	Glioblastoma IDH mutant G4	+	+	+	—	+	43

[1] Genetic tests were not performed in these patients, who were diagnosed before implementation of genetic testing. Retrospective testing was negative.

[2] A lack of necessary molecular information.
