# Supplementary material for: Combining amino acid PET and MRI imaging increases accuracy to define malignant areas in adult glioma
Source: Nat Commun. 2023 Jul 29;14:4572. doi: 10.1038/s41467-023-39731-8 (PMC10387066; doi:10.1038/s41467-023-39731-8)
Supplement: Supplementary file 3 — Reporting Summary [file 41467_2023_39731_MOESM3_ESM.pdf]

nature portfolio

Corresponding author(s): Maciej Harat

Last updated by author(s): 24/05/2023

Reporting Summary

Nature Portfolio wishes to improve the reproducibility of the work that we publish. This form provides structure for consistency and transparency in reporting. For further information on Nature Portfolio policies, see our Editorial Policies and the Editorial Policy Checklist.

Statistics

For all statistical analyses, confirm that the following items are present in the figure legend, table legend, main text, or Methods section.

n/a	Confirmed	
☐	☒	The exact sample size (*n*) for each experimental group/condition, given as a discrete number and unit of measurement
☐	☒	A statement on whether measurements were taken from distinct samples or whether the same sample was measured repeatedly
☐	☒	The statistical test(s) used AND whether they are one- or two-sided<br>*Only common tests should be described solely by name; describe more complex techniques in the Methods section.*
☐	☒	A description of all covariates tested
☐	☒	A description of any assumptions or corrections, such as tests of normality and adjustment for multiple comparisons
☐	☒	A full description of the statistical parameters including central tendency (e.g. means) or other basic estimates (e.g. regression coefficient) AND variation (e.g. standard deviation) or associated estimates of uncertainty (e.g. confidence intervals)
☐	☒	For null hypothesis testing, the test statistic (e.g. *F*, *t*, *r*) with confidence intervals, effect sizes, degrees of freedom and *P* value noted<br>*Give P values as exact values whenever suitable.*
☒	☐	For Bayesian analysis, information on the choice of priors and Markov chain Monte Carlo settings
☒	☐	For hierarchical and complex designs, identification of the appropriate level for tests and full reporting of outcomes
☐	☒	Estimates of effect sizes (e.g. Cohen's *d*, Pearson's *r*), indicating how they were calculated

Our web collection on statistics for biologists contains articles on many of the points above.

Software and code

Policy information about availability of computer code

Data collection	All imaging data were collected on a Siemens Biograph mMR dual modality PET/MR scanner and Brainlab software (iPlan Stereotaxy 3.0). The PET-MR analysis conducted prior to biopsy was fully quantitative using dedicated software (Syngovia VB60, Siemens Healthcare, Erlangen, Germany).
Data analysis	All analyses were conducted in PQStat v.1.8 (PQStat Software, Poznan, Poland).

For manuscripts utilizing custom algorithms or software that are central to the research but not yet described in published literature, software must be made available to editors and reviewers. We strongly encourage code deposition in a community repository (e.g. GitHub). See the Nature Portfolio guidelines for submitting code & software for further information.

Data

Policy information about availability of data

All manuscripts must include a data availability statement. This statement should provide the following information, where applicable:

- Accession codes, unique identifiers, or web links for publicly available datasets
- A description of any restrictions on data availability
- For clinical datasets or third party data, please ensure that the statement adheres to our policy

All data generated in this study are provided in the Supplementary Information/Source Data file

Research involving human participants, their data, or biological material

Policy information about studies with human participants or human data. See also policy information about sex, gender (identity/presentation), and sexual orientation and race, ethnicity and racism.

Reporting on sex and gender	Findings apply to both sexes (M 11 patients, F 12 patients), and sex and gender were not considered in study design. Sex was determined by self-reporting and collected in the source data. Consent was obtained for sharing of individual-level data. Overall, sex and gender analyses were not performed, as in all studied cases, high FET uptake was visualized and confirmed outside areas of contrast enhancement on MRI, indicative of high-grade glioma.
Reporting on race, ethnicity, or other socially relevant groupings	In Poland, White ethnicity is far more frequent than other ethnicities. This epidemiological factor likely skewed the demographic profile of the studied group, and any additional differences in demographic characteristics from what has been observed at a global population level could be attributed to sampling randomness.
Population characteristics	The patient group consisted of male and female subjects diagnosed with brain tumors in MRI. The mean age of patients in our group was 48, range 18-86, mean Karnofsky performance status 90 (range 70-100). 6 patients were previously treated with radiotherapy, and 17 patients were untreated. The inclusion criteria were: age >18 years; suspected CNS glioma based on MRI; and in general good health permissive of surgery.
Recruitment	All patients were recruited by neurosurgeons from patients attending the Department of Neurosurgery, 10th Military Research Hospital, Bydgoszcz, Poland for stereotactic biopsy due to suspected primary brain tumors of glial origin based on contrast-enhanced MRI and symptoms. The total number of patients enrolled was 23.<br>All patients provided written informed consent to participate based on the principles of the Declaration of Helsinki.<br>Since the study was conducted at a single site, there is a bias to recruiting patients who either live near the hospital or who are willing to travel long distances to receive care at the nationally-recognized neurosurgery department. In addition, in Poland, White ethnicity is far more frequent than other ethnicities. This epidemiological factor likely skewed the demographic profile of the studied group, and any additional differences in demographic characteristics from what has been observed at a global population level could be attributed to sampling randomness.
Ethics oversight	The institution's bioethics committee of Collegium Medicum Nicolaus Copernicus University approved the study (KB 647/2015), and informed consent was obtained from all participants,

Note that full information on the approval of the study protocol must also be provided in the manuscript.

Field-specific reporting

Please select the one below that is the best fit for your research. If you are not sure, read the appropriate sections before making your selection.

[x] Life sciences [ ] Behavioural & social sciences [ ] Ecological, evolutionary & environmental sciences

For a reference copy of the document with all sections, see nature.com/documents/nr-reporting-summary-flat.pdf

Life sciences study design

All studies must disclose on these points even when the disclosure is negative.

Sample size	The sample was selected to cover the different stages of adulthood, both sexes and irradiated or non-treated patients, and efforts were made to ensure comparable representation of each sex. The total sample size was selected based power estimates from prior developmental studies. All participants reported no history of psychiatric or other neurological diseases.
Data exclusions	The exclusion criteria were: age <18 years; KPS <60 points; pregnancy; and disqualification from surgery for medical reasons.
Replication	Replication of individual tissue sample analysis was not performed (as per histopathological protocol); however, efforts were made to verify reproducibility by serial biopsy assessment and overall patient numbers. Several tumor samples per patient per target were evaluated, and all results are presented in the source data. All techniques and reagents used for the analyses of this study had previously been optimized and validated in clinic. In general, please note that technical replicates of tumor biopsies were not performed due to limited sample volumes. Presented photomicrographs were double checked by a pathologist blinded to the case.
Randomization	Randomization was not relevant to this study. Participants were a cohort of the same group: adult patients with brain tumor suspected of glioma.
Blinding	Blinding was not relevant to this study. Patients were not allocated into experimental groups and treatments were planned on a patient-by-patient basis.

Reporting for specific materials, systems and methods

We require information from authors about some types of materials, experimental systems and methods used in many studies. Here, indicate whether each material, system or method listed is relevant to your study. If you are not sure if a list item applies to your research, read the appropriate section before selecting a response.

Materials & experimental systems

n/a	Involved in the study
☒	☐ Antibodies
☒	☐ Eukaryotic cell lines
☒	☐ Palaeontology and archaeology
☒	☐ Animals and other organisms
☒	☐ Clinical data
☒	☐ Dual use research of concern
☒	☐ Plants

Methods

n/a	Involved in the study
☒	☐ ChIP-seq
☒	☐ Flow cytometry
☐	☒ MRI-based neuroimaging

Magnetic resonance imaging

Experimental design

Design type: Structural neuroimaging

Design specifications: T1-weighted (T1W with and without contrast agent), T2-weighted (T2W), fluid-attenuated inversion recovery (FLAIR)

Behavioral performance measures: No behavioral tasks were performed.

Acquisition

Imaging type(s): Structural

Field strength: 3 Tesla

Sequence & imaging parameters: TlW and T2W images were acquired with 2D FSE (TlW: TR/TE = 2700/25 ms, inversion time = 830 ms, FA= 111°, ETL = 8, acquisition matrix = 340x280, FOY = 230x230 mm 2, acquisition slice thickness/slice gap = 1/0.5 mm, 27 slices, and NEX = 1; T2W: TR/TE = 5900/106 ms, FA = 120°, ETL = 30, acquisition matrix = 448x448, FOY = 230x230 mm2, acquisition slice thickness/slice gap = 5/0.5 mm, 27 slices, and NEX= 2). FLAIR images were acquired with 3D FSE with TR/TE = 6300/104 ms, inversion time : 1800 ms, FA = 90/180°, ETL = 180, acquisition matrix = 256x256x60, FOY= 250x250x150 mm3, acquisition slice thickness = 2.5 mm, and NEX = 1.

Area of acquisition: Brain

Diffusion MRI ☐ Used ☒ Not used

Preprocessing

Preprocessing software: No preprocessing procedure was used

Normalization: No normalization procedure was used

Normalization template: No normalization template was used

Noise and artifact removal: No artifact removal procedures were performed

Volume censoring: No custom image masks were used for any image processing steps

Statistical modeling & inference

Model type and settings: No statistical modeling and inference were used

Effect(s) tested: No tasks or stimulus effects were tested

Specify type of analysis: ☐ Whole brain ☐ ROI-based ☐ Both

Statistic type for inference (See Eklund et al. 2016): No statistical modeling and inference were used

Correction: No statistical modeling and inference were used

Models & analysis

n/a	Involved in the study
☒	☐ Functional and/or effective connectivity
☒	☐ Graph analysis
☒	☐ Multivariate modeling or predictive analysis
